# Supplementary material for: Priming of dendritic cells by DNA-containing extracellular vesicles from activated T cells through antigen-driven contacts
Source: Nat Commun. 2018 Jul 9;9:2658. doi: 10.1038/s41467-018-05077-9 (PMC6037695; doi:10.1038/s41467-018-05077-9)
Supplement: Supplementary file 1 — Supplementary Information [file 41467_2018_5077_MOESM1_ESM.pdf]

Priming of dendritic cells by DNA-containing extracellular vesicles from activated T cells through antigen-driven contacts

Torralba et al.

Supplementary information

Supplementary Figure 1

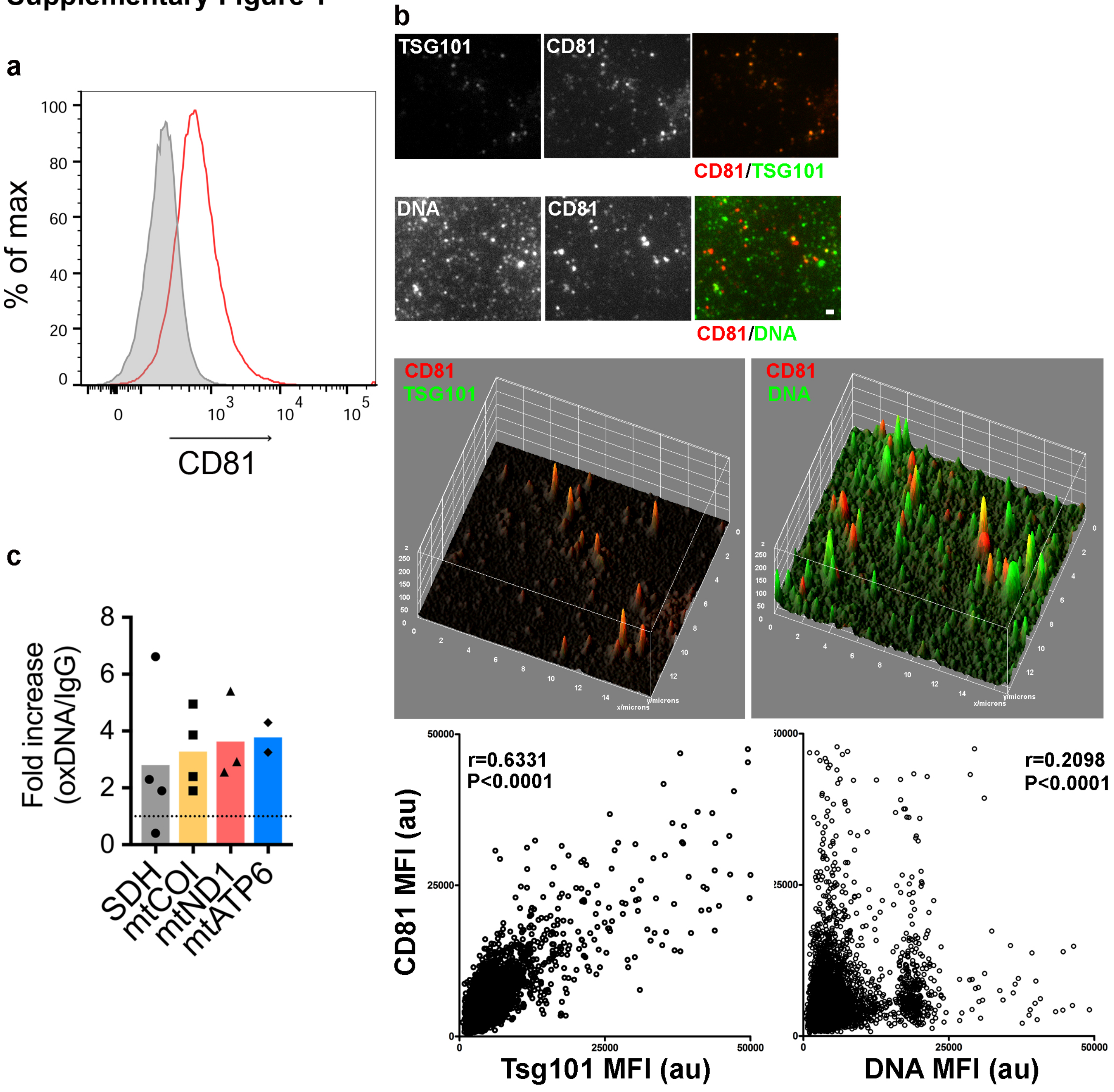

Supplementary Figure 1.

a) Exosomes from primary mouse T lymphoblasts were purified, coupled to aldehyde sulfate beads and analyzed by flow cytometry by staining with the anti-CD81 Ab under non-permeabilizing conditions. Histograms show CD81 staining in exosomes coupled to aldehyde sulfate beads. b) Images from isolated exosomal preparations stained for CD81 (red in merged images) and TSG101 or DNA (green in merged images). Exosomal preparations were spun on coverslips, fixed, processed for immunofluorescence and imaged in parallel. Permeabilization was performed upon CD81 staining. Imaging was performed with a TIRFm with a laser penetrance of 90 nm. Individual spots were detected. Scale bar, 1 µm. Graphs, Qualitative co-localization of fluorescence (yellow in merged images and graphs). The fluorescence intensity is plotted versus xy localization of the merged images showed. Scatter plots, correlation between mean fluorescence intensity from CD81:Tsg101 or CD81:DNA from TIRF images (r, Spearman coefficient for correlation; $P<0.001$, $n=5937$). Graphs were obtained with the Interactive 3D Surface Plot Plugging from Image J (https://imagej.nih.gov/ij/plugins/surface-plot-3d.html). Scatter plots correspond to the data from analysis with Image J from 20 different images from two different exosomal preparations from mouse T lymphoblasts. Images were normalized and processed in parallel. **c)** PCR analysis of the enrichment of the indicated mitochondrial (mtCO1, mtND1, mtATP6) or genomic (succinate dehydrogenase, SDH) genes in the DNA obtained from exosomes and immunoprecipitated with 8-OHdG Ab relative to isotype control IgG. Mean from quantification of 4 independent experiments.

Supplementary Figure 2

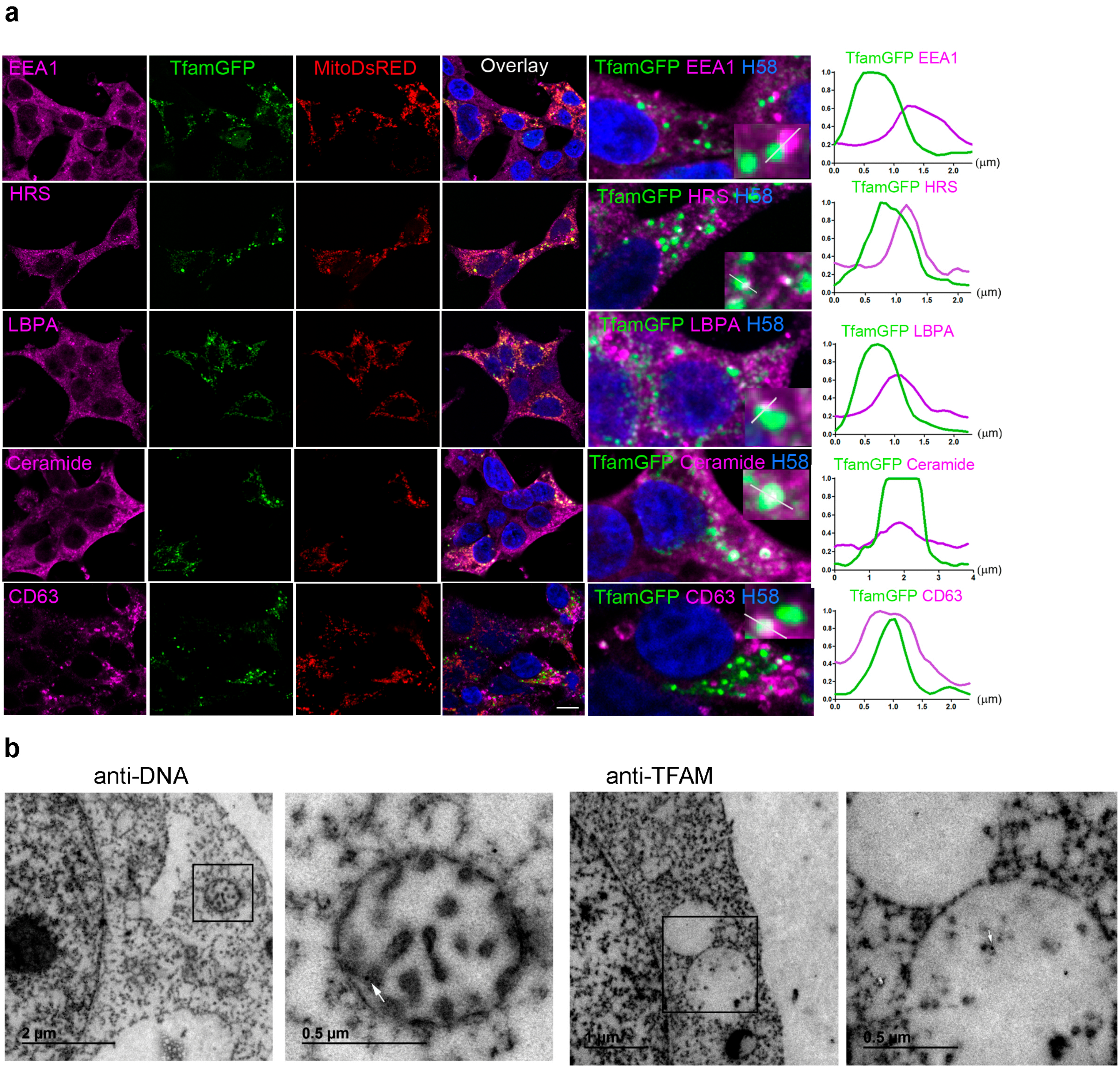

Supplementary Figure 2.

a) Confocal co-localization analysis in HEK293 cells co-transfected with TFAM-GFP (green), nuclei with HOECHST 58 (blue) and a mitochondrial targeted fluorescent protein (mitoDsRed, red) and immunostained for endolysosomal compartment markers: the early endosome marker EEA1 and the ESCRT components HRS, LBPA, CD63, and ceramide (purple). Right images show high magnification views of co-localization between TFAM-GFP (green) and the various endolysosomal markers (purple). Charts show the fluorescence profiles along the corresponding white lines in the inset panels. Bar, 10 µm **b)** Representative electron microscopy images showing DNA and TFAM immunogold staining in canonical multivesicular bodies from HEK293T cells transfected with the active mutant Rab7-Q67L-GFP.

Supplementary Figure 3

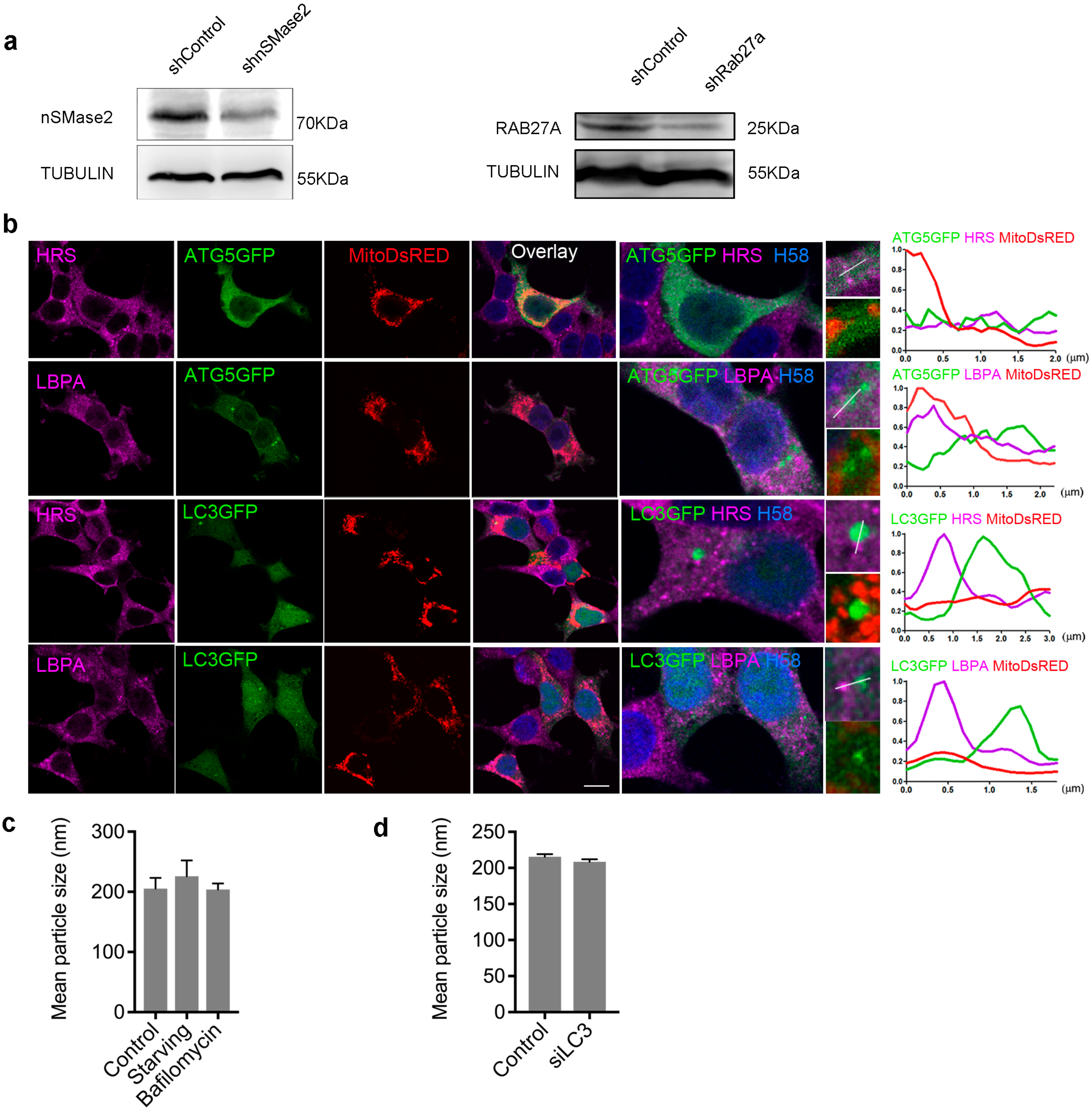

Supplementary Figure 3

a) Left, Western blot analysis of nSMase 2 knockdown in J77 T cells infected with shControl or shnSMase2 shRNA. Right, Western blot analysis of Rab27a knockdown in HEK293 cells infected with specific shControl or shRab27a RNA. **b)** Confocal co-localization analysis in HEK293 cells co-transfected with ATG5-GFP or LC3-GFP (green) and a mitochondria-targeted fluorescent protein (mitoDsRed, red) and stained with the endolysosomal compartment markers HRS and LBPA (purple) and nuclei with HOECHST 58 (blue). Right images show high magnification views of co-localization between ATG5/LC3 (green) and the various endo-lysosomal markers (purple). Charts show the fluorescence profiles along the corresponding white lines in the adjacent panels. Bar, 10 µm. **c)** Size distribution analysis by Nanoparticle Tracking Analysis (NTA) of purified exosomes obtained from equal numbers of Jurkat T cells left untreated, serum-starved overnight, or treated with bafilomycin A. **d)** Size distribution analysis by Nanoparticle Tracking Analysis (NTA) of purified exosomes obtained from equal numbers of Jurkat T cells transfected with control siRNA or siRNA targeting LC3 from two independent experiments.

Supplementary Figure 4

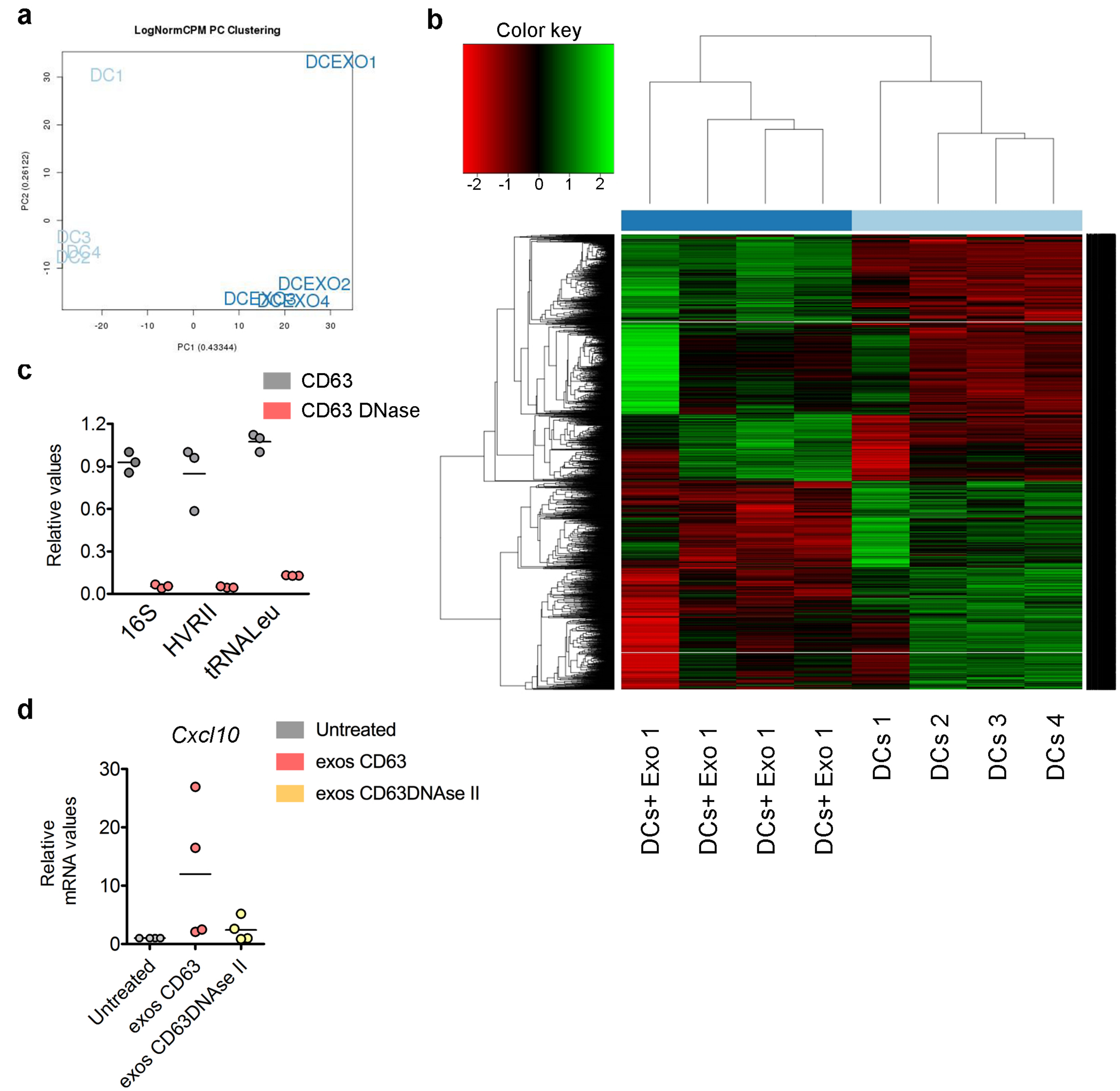

Supplementary Figure 4

a) Principal Component Analysis (PCA) of the RNA samples obtained from 4 biological replicates of DCs and DCs incubated with exosomes. **b)** Heatmap for the differently regulated genes in 4 biological replicates of DCs left untreated or treated with exosomes. **c)** The level of mtDNA assessed by PCR amplification in EVs obtained from the culture supernatant of human HEK293 cells overexpressing CD63GFP or CD63 fused to DNase II. Mean. n=3. **d)** qRT-PCR analysis of *Cxcl10* in hDCs upon addition of exosomes from CD63GFP or CD63GFP-DNase II overexpressing HEK293 cells. Data show four independent experiments obtained from the culture supernatant of human HEK293 cells overexpressing CD63GFP or CD63 fused to DNase II. Mean. n=4.

Supplementary Figure 5

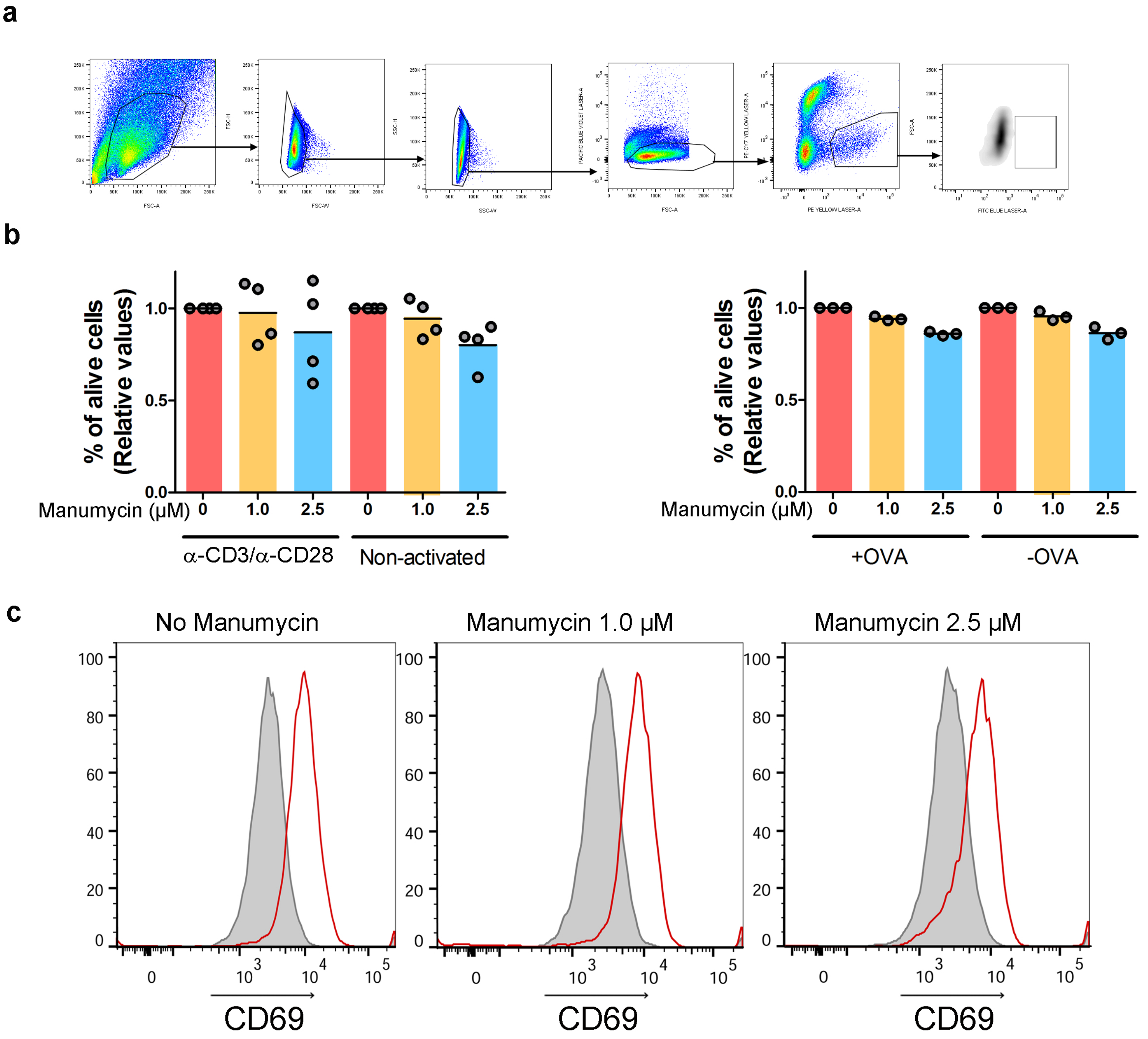

Supplementary Figure 5

a) Gating strategy for Figure 6d. Last dot plot corresponds to the represented in figure. **b)** Left graph, Percentage of alive primary T lymphoblasts treated with manumycin for 16 h (vehicle = 0, 1 and 2.5 µM) and concomitant activation with anti-CD3 and anti-CD28 monoclonal Abs. Cell survival was measured by FACs, based on exclusion of DAPI staining. All values are relative to survival of non-treated cells. Mean percentage of T cell death was 13 % at maximal manumycin concentration. Data represent four independent experiments. Right graph, Percentage of alive primary T lymphoblasts upon pre-treatment with increasing concentrations of manumycin for 2 h (vehicle = 0, 1 and 2.5 µM) and activation with OVA-pulsed dendritic cells for an additional 4 h period at a 5:1 ratio T cell: DCs. Manumycin was present during activation. Cell survival was measured by FACs, based on exclusion of DAPI staining. All values are relative to survival of non-treated cells. Mean percentage of T cell death was 13.8 % at maximal manumycin concentration. Data represent three independent experiments. **c)** Cells treated as in b) (CD3-CD28) were stained with anti-CD69 as a measure of T cell activation. Histograms show a representative experiment out of four. No significant changes were observed in CD69 levels.

Supplementary Figure 6

Figure 1d

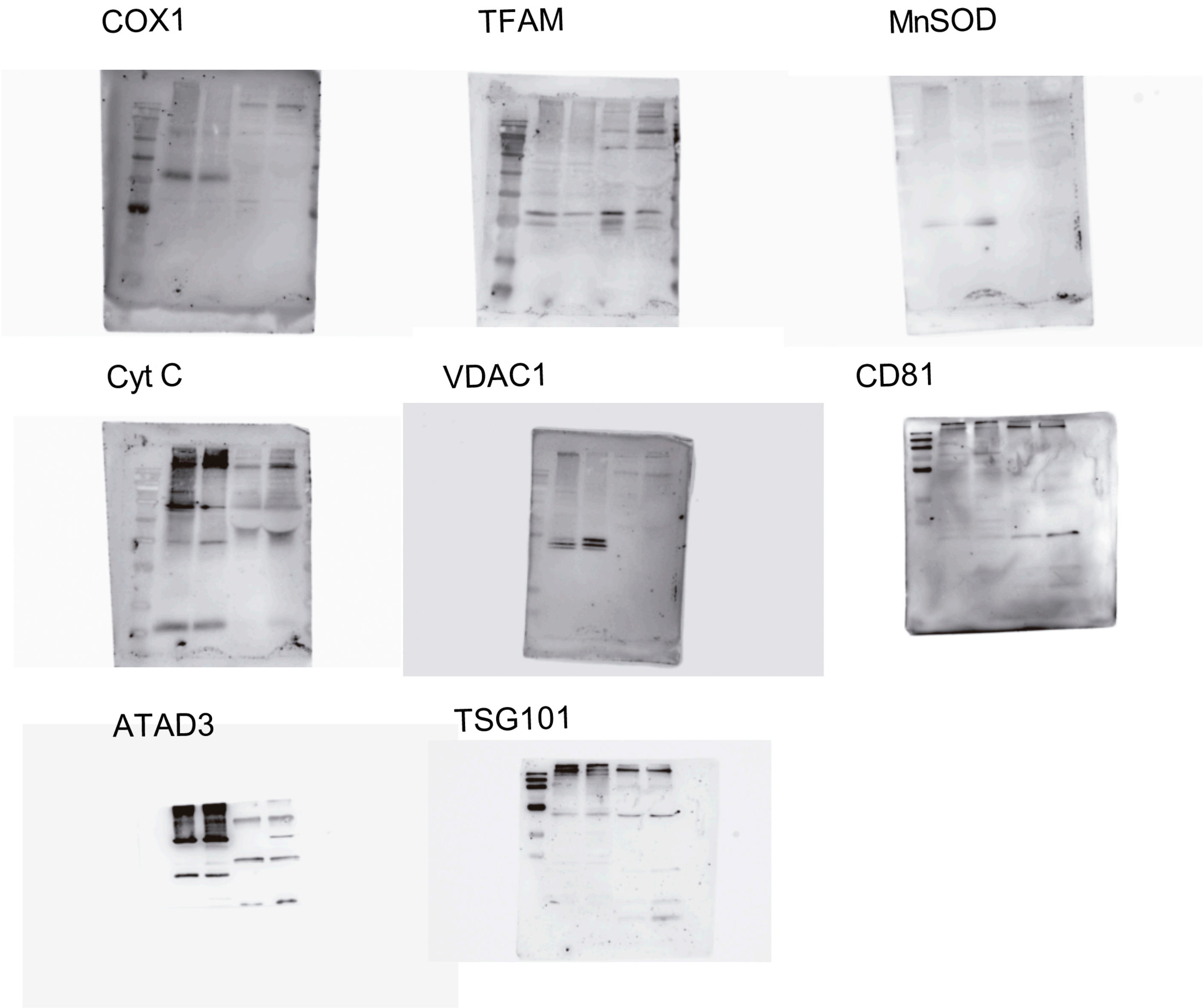

Supplementary Figure 6. Uncropped western blots from Figure 1d

Supplementary Figure 7

Figure 1e

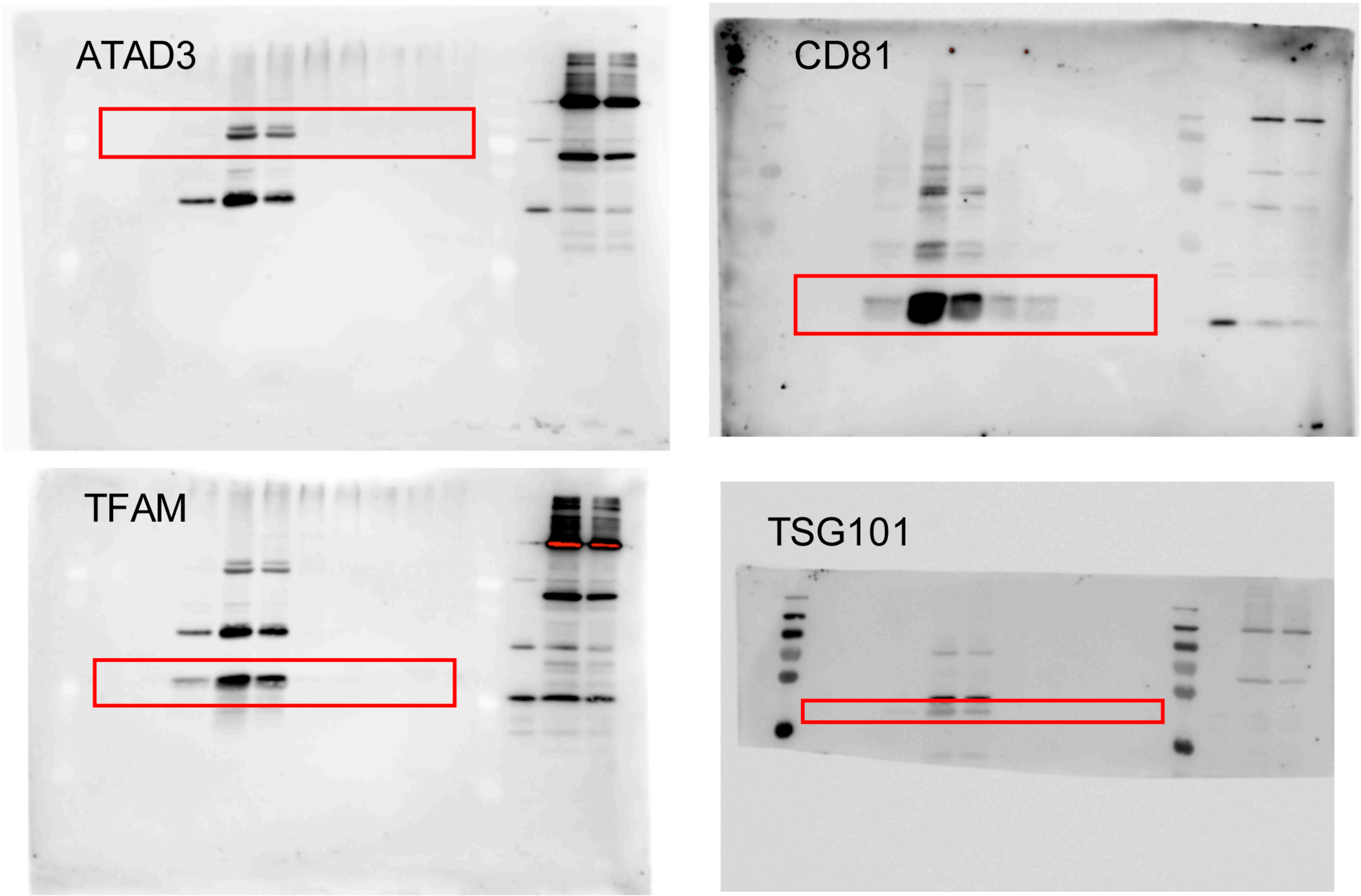

Supplementary Figure 7. Uncropped western blots from Figure 1e

Supplementary Figure 8

Figure 2b

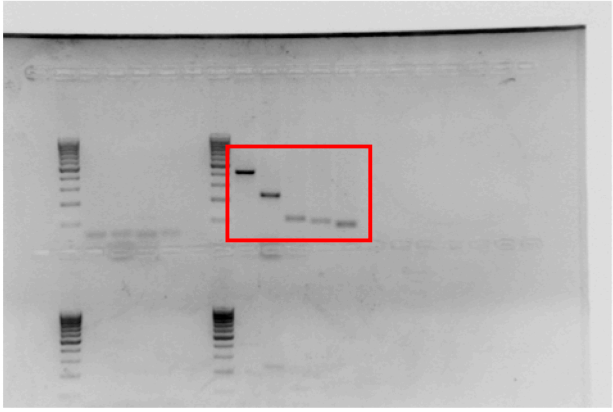

Figure 2c

HVRII (PCR)

ß2 microglobulin (PCR)

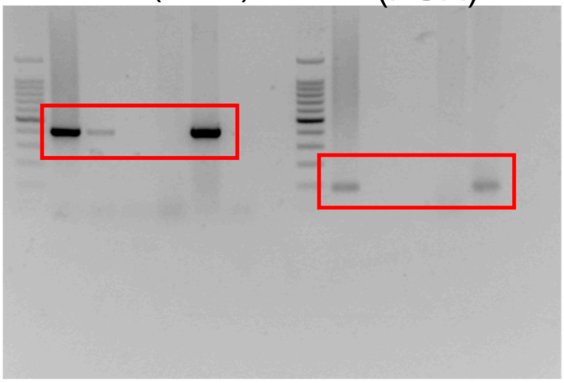

Figure 2d

TSG101

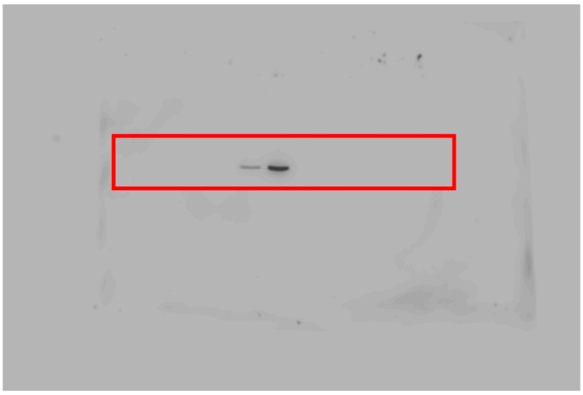

CD63

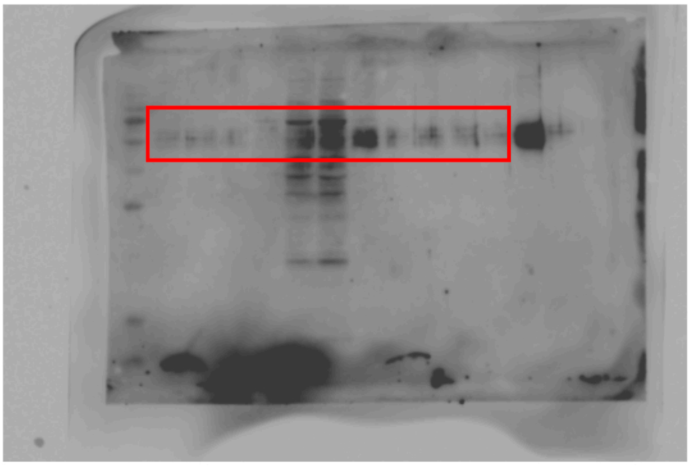

HVRII (PCR)

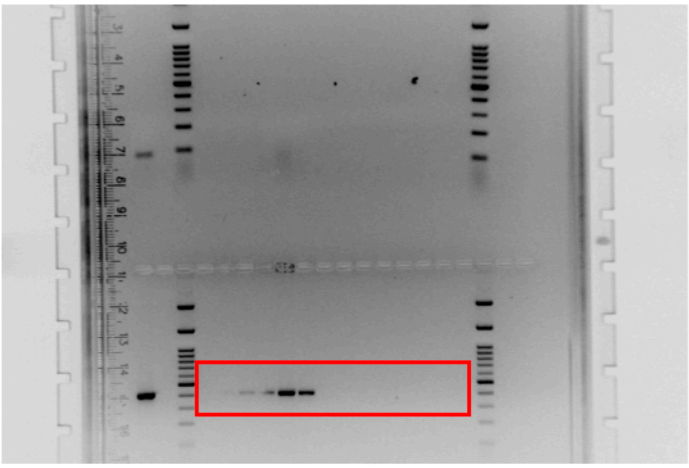

Supplementary Figure 8. Uncropped western blots and DNA electrophoresis from Figure 2 as indicated.

Supplementary Figure 9

Figure 3c

TFAM

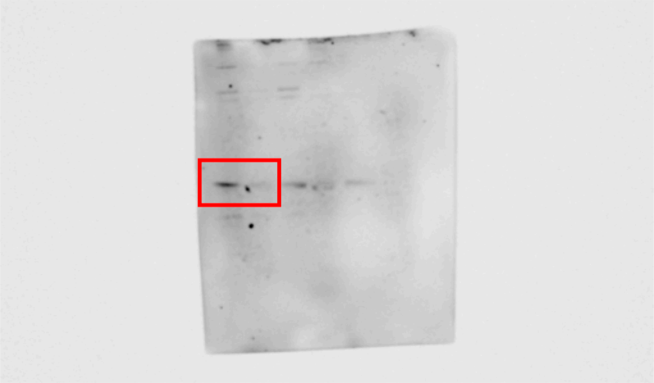

ATAD

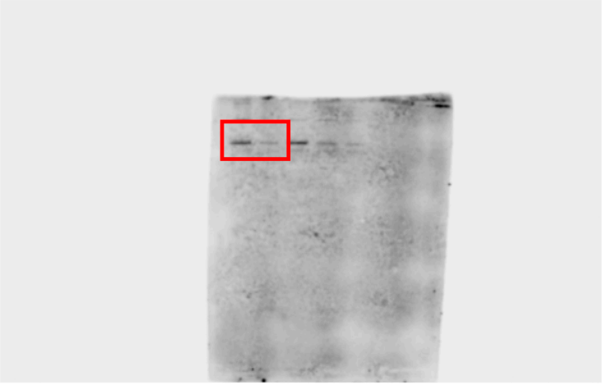

CD63

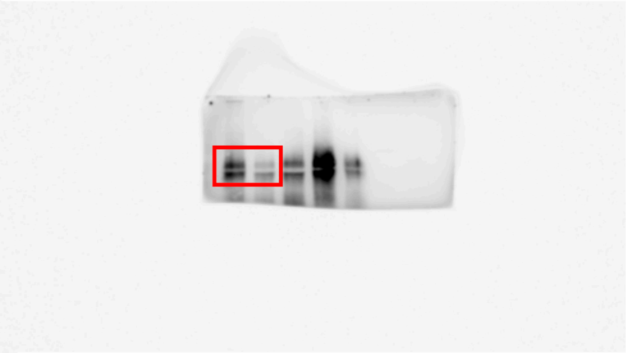

HVRII (PCR)

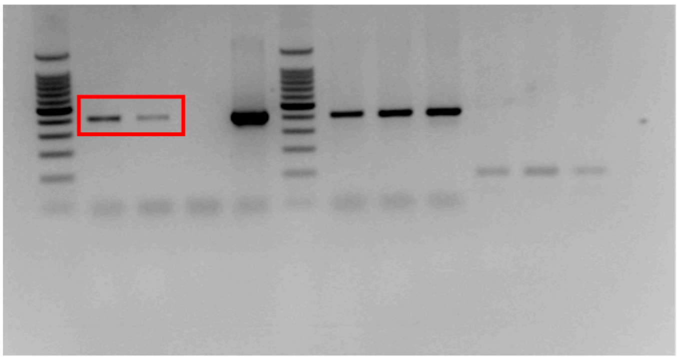

Figure 3g

TFAM

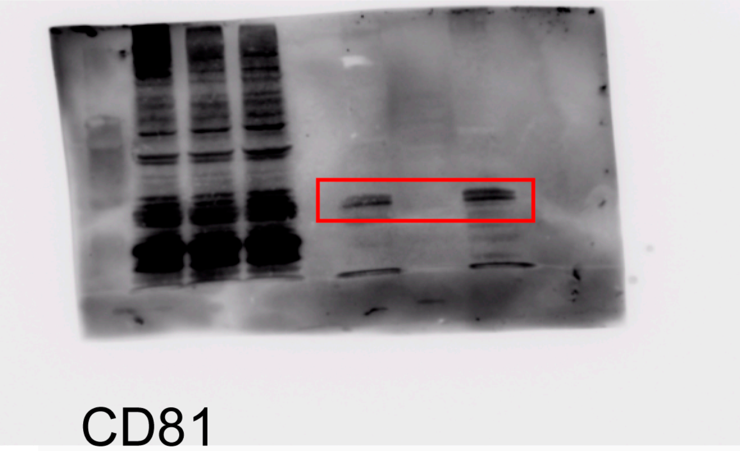

CD63

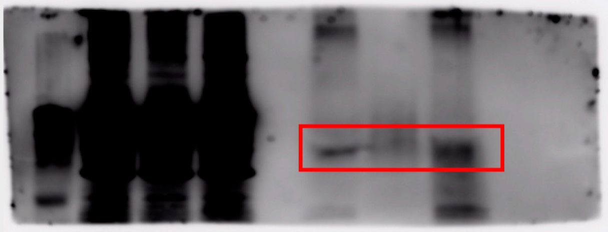

CD81

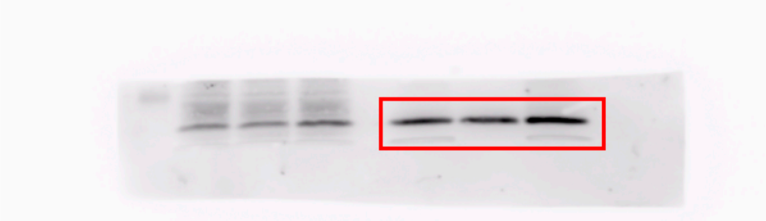

Supplementary Figure 9. Uncropped western blots and DNa electrophoresis from Figure 3 as indicated.

Supplementary Figure 10

Figure 3h

TFAM

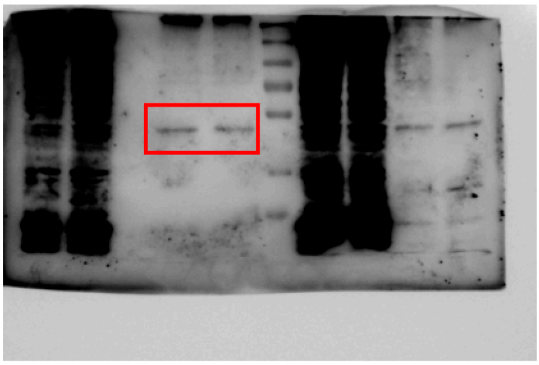

CD63

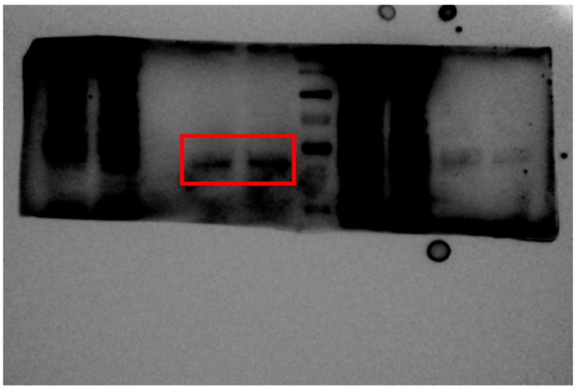

CD81

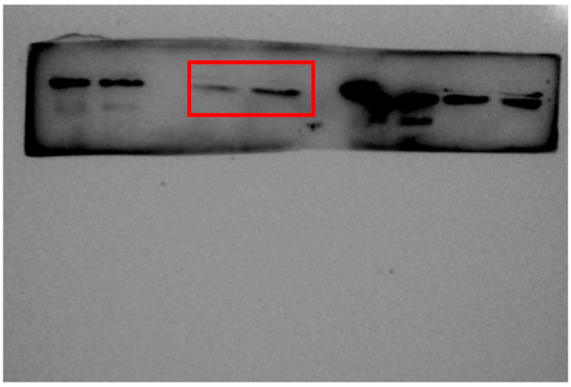

Supplementary Figure 10. Uncropped western blots from Figure 3h

Supplementary Figure 11

Figure 4b

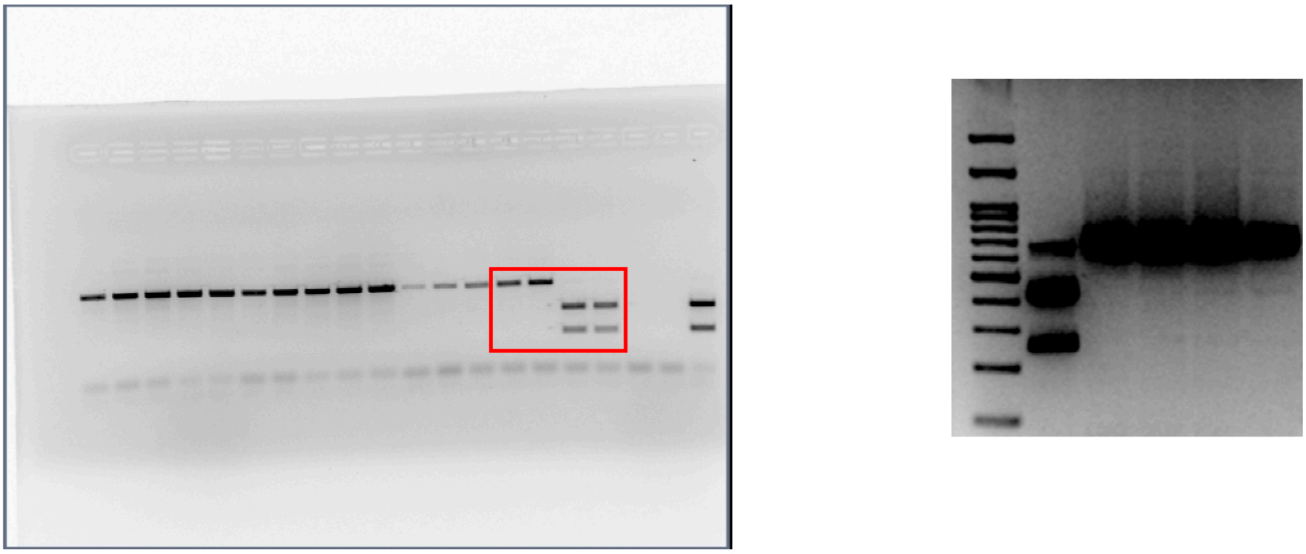

Figure 4e

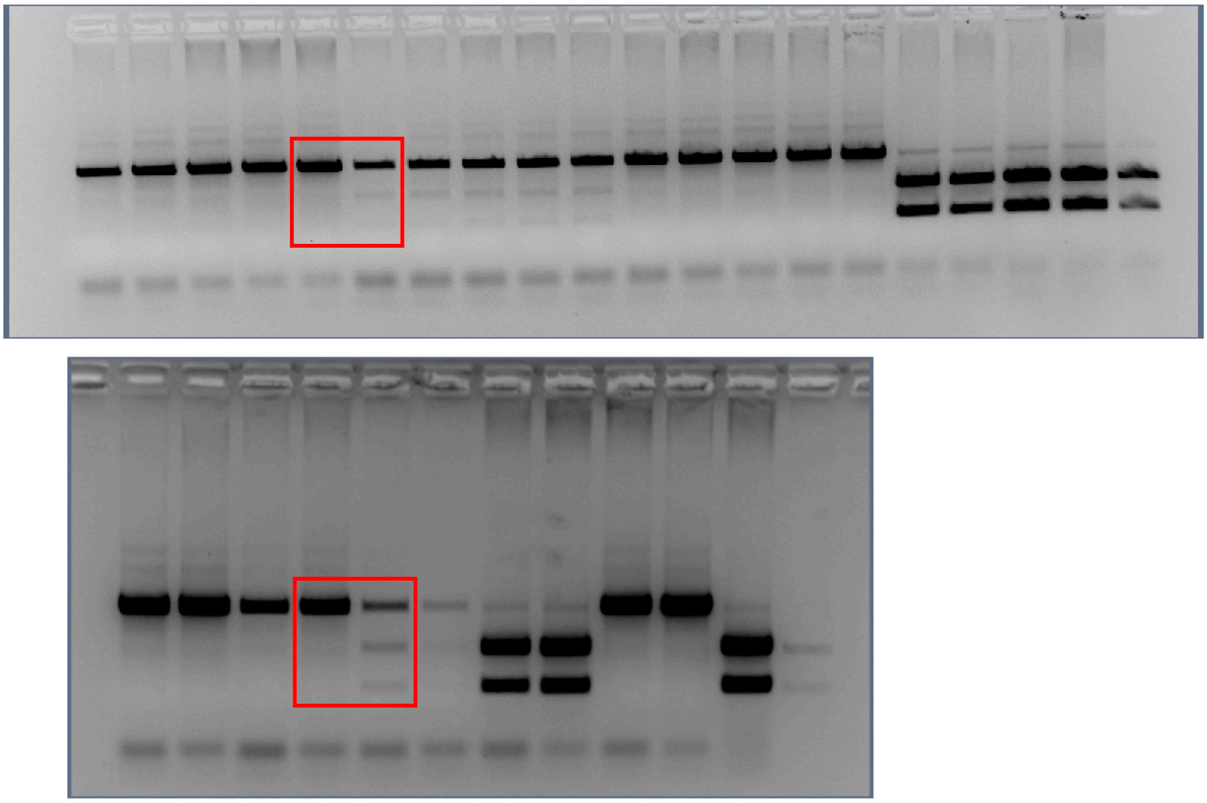

Figure 4f

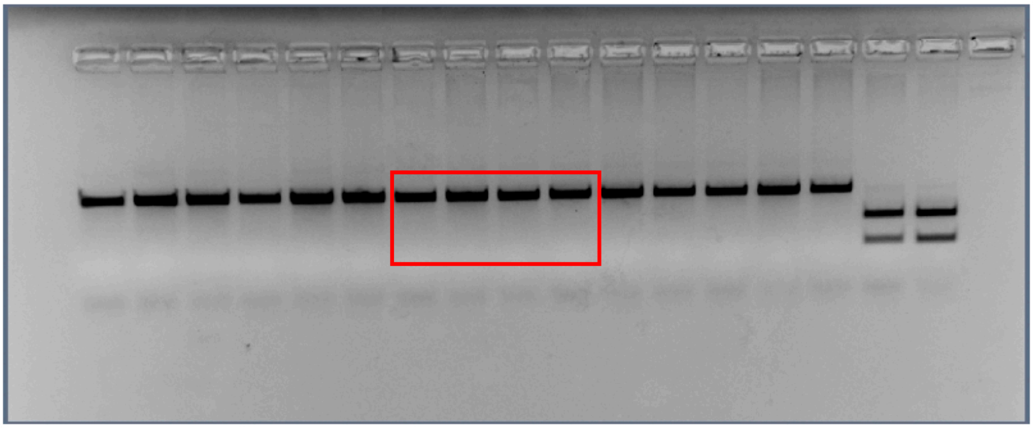

Supplementary Figure 11. Uncropped DNA electrophoresis images from Figure 4 as indicated.

Supplementary Table 1. Primers for qPCR

	Sequence	gene	Organism
Forward	CCAGTGCTGCCGTCATTTTC	*Cxl10*	Mouse
Reverse	GGCTCGCAGGGATGATTTCAA	*Cxl10*	Mouse
Forward	CTAGAGCTAGAGCCTGCAG	*Isg15*	Mouse
Reverse	AGTTAGTCACGGACACCAG	*Isg15*	Mouse
Forward	GAGAGGACCATGAAGAGGA	*Usp18*	Mouse
Reverse	TAAACCAACCAGACCATGAG	*Usp18*	Mouse
Forward	TTCCCAGCAGCACAGAAAC	*Ifit3*	Mouse
Reverse	AAATTCCAGGTGAAATGGCA	*Ifit3*	Mouse
Forward	CAAGGCAGGTTTCTGAGGAG	*Ifit1*	Mouse
Reverse	GACCTGGTCACCATCAGCAT	*Ifit1*	Mouse
Forward	CGCGCATGCAACTGGCATATAACT	*Stat1*	Mouse
Reverse	ATGCTTCCGTTCCCACGTAGACTT	*Stat1*	Mouse
Forward	AGCAGAGGAACCTCCAGTCT	*CXCL10*	Human
Reverse	ATGCAGGTACAGCGTACAGT	*CXCL10*	Human

Supplementary Table 2. Primers for PCR

	Sequence	gene	Organism
Forward	CACCCAAGAACAGGGTTTGT	*tRNA Leu*	Human
Reverse	TGGCCATGGGTATGTTGTTA	*tRNA Leu*	Human
Forward	GCCTTCCCCCGTAAATGATA	*16S RNA*	Human
Reverse	TTATGCGATTACCGGGCTCT	*16S RNA*	Human
Forward	TGCTGTCTCCATGTTTGATGTATCT	*B2M*	Human
Reverse	TCTCTGCTCCCCACCTCTAAGT	*B2M*	Human
Forward	TTCGGCGCATGAGCTGGAGTCC	*hCOX*	Human
Reverse	TATGCGGGGAAACGCCATATCG	*hCOX*	Human
Forward	CTCACGGGAGCTCTCCATGC	*HVRII*	Human
Reverse	CTGTTAAAAGTGCATACCGCCA	*HVRII*	Human
Forward	ACCCCTTCTCTGTCTACCG	*SDH*	Mouse
Reverse	AATGCTCGCTTCTCCTTGTAG	*SDH*	Mouse
Forward	CCCAGATATAGCATTCCCACG	*mtCOXI*	Mouse
Reverse	ACTGTTCATCCTGTTCCTGC	*mtCOXI*	Mouse
Forward	TGCACCTACCCTATCACTCA	*mtND1*	Mouse
Reverse	GGCTCATCCTGATCATAGAATGG	*mtND1*	Mouse
Forward	TCCCAATCGTTGTAGCCATC	*mtATP6*	Mouse
Reverse	TGTTGGAAAGAATGGAGTCGG	*mtATP6*	Mouse

Supplementary References

1. Tabas-Madrid, D., Nogales-Cadenas, R., and Pascual-Montano, A. (2012). GeneCodis3: a non-redundant and modular enrichment analysis tool for functional genomics. Nucleic Acids Res *40*, W478-483.
