# Supplementary material for: Priming of dendritic cells by DNA-containing extracellular vesicles from activated T cells through antigen-driven contacts
Source: Nat Commun. 2018 Jul 9;9:2658. doi: 10.1038/s41467-018-05077-9 (PMC6037695; doi:10.1038/s41467-018-05077-9)
Supplement: Supplementary file 3 — Description of Additional Supplementary Files [file 41467_2018_5077_MOESM3_ESM.pdf]

Description of Additional Supplementary Files

File Name: Supplementary Data 1
Description: High-throughput mass spectrometry analysis of proteins from exosomes. Number of unique peptides of proteins that were identified in exosomes samples isolated from mouse T lymphoblasts. Four biological replicates of proteins extracts of exosomes were trypsin digested and analyzed by LC-MS/MS. The table represent the number of peptides identified at 1% of FDR corresponding to proteins identified with 2 unique peptides and/or in at least 2 replicates.

File Name: Supplementary Data 2
Description: Exosomal proteins related to mitochondrial localization and function identified by Gene Ontology (GO) enrichment analysis. List of proteins identified in exosomes samples isolated from four biological replicates of protein extracts of exosomes. Identified proteins were analyzed by Gene Ontology (GO) of cellular component with GeneCodis 3.0.
